# *IDH1* R132C mutation is detected in clear cell hepatocellular carcinoma by pyrosequencing

**DOI:** 10.1186/s12957-017-1144-1

**Published:** 2017-04-12

**Authors:** Jung Hee Lee, Dong Hoon Shin, Won Young Park, Nari Shin, Ahrong Kim, Hyun Jung Lee, Young Keum Kim, Kyung Un Choi, Jee Yeon Kim, Young Il Yang, Chang Hun Lee, Mee Young Sol

**Affiliations:** 1 Department of Pathology, School of Medicine, Pusan National University, Yangsan, 50612 South Korea; 2 Research Institute for Convergence of Biomedical Science and Technology, Pusan National University Yangsan Hospital, Yangsan, 50612 South Korea; 3 Paik Institute for Clinical Research, Inje University College of Medicine, Busan, 47392 South Korea

**Keywords:** IDH, Hepatocellular carcinoma, Clear cell carcinoma, Pyrosequencing

## Abstract

**Background:**

*Isocitrate dehydrogenase 1* (*IDH1*) mutation is common in low-grade glioma (approximately 80%) and acute myeloid leukemia (approximately 10%). Other than brain tumor or hematologic malignancies, intrahepatic cholangiocarcinoma (iCC) is a well-known solid tumor with *IDH1* mutation (6.8–20%). Histologically, poor differentiation and clear cell change are associated with *IDH1* mutation in iCC. Since hepatocellular carcinoma (HCC) shares histologic features with iCC, some specific subtypes of HCC might show a higher *IDH1* mutation rate than reported before (0.5–1.5%).

**Methods:**

Forty-six cases of iCC and 48 cases of HCC (including 20 cases of clear cell type and 13 cases of pseudoglandular pattern) were tested for *IDH1* mutation by pyrosequencing.

**Results:**

Three cases in iCC (6.5%) and five cases in HCC (10.4%) had *IDH1* mutation, all of which were Arg132Cys. *IDH1* mutant HCCs were all clear cell type. Although the *IDH1* mutation rate between iCC and HCC demonstrated no significant difference, clear cell HCC revealed statistically increased mutation rate compared to that of HCC without clear cell change (*P* = 0.009). Presence of *IDH1* mutation was related with poor survival in clear cell HCC patients (*P* = 0.004).

**Conclusions:**

Clear cell HCC showed higher frequency of *IDH1* mutation rate than other variants of HCC. This result consolidates the assumption that morphological features of tumors reflect molecular alterations.

## Background

The *isocitrate dehydrogenase 1* (*IDH1*) gene encodes isocitrate dehydrogenase 1 which is an isoform of IDH, a key enzyme of tricarboxylic acid (TCA) cycle. IDH catalyzes the oxidative decarboxylation of isocitrate to α-ketoglutarate using nicotinamide adenine dinucleotide phosphate (NADPH) as the electron acceptor. IDH1 is highly expressed in the liver, localized in the cytoplasm, and involved in lipid metabolism and glucose sensing [1].

*IDH1* gene mutations have been widely studied in glioma or leukemia patients [2]. The major alteration observed in mutant *IDH1* gene is the substitution of arginine at codon 132. Wild type *IDH1* Arg132 is a critical binding point for the isocitrate substrate. The mutant IDH1 protein has increased affinity for NADPH, promoting the reduction of α-ketoglutarate to d-2-hydroxyglutarate. The mechanism leading to carcinogenesis due to *IDH1* mutations needs to be elucidated, but it has been suggested that d-2-hydroxyglutarate plays a role [3].

Other solid tumors rarely show *IDH1* mutations [4, 5]. Unexpectedly, some intrahepatic cholangiocarcinomas (iCCs) presented with *IDH1* mutations [6]. The mutation rate of *IDH1* in iCCs has been reported between 6.8 and 20% [6–8]. It is noteworthy that among the carcinomas of the digestive system, only iCCs showed significantly increased *IDH1* mutation rates [8].

An iCC is an anatomical subtype of a cholangiocarcinoma. Perihilar and extrahepatic cholangiocarcinomas are the other two anatomical subtypes. These three subtypes share their cellular origin, “the bile duct epithelium,” but *IDH1* mutations are rarely observed in latter two subtypes [6]. Meanwhile, hepatocellular carcinomas (HCCs) have their anatomic location in common with iCCs. An HCC is a heterogeneous tumor, which occasionally makes it difficult to differentiate from iCCs radiologically, macroscopically, or microscopically. Hence, we supposed that if HCCs have some overlapped histological features with iCCs, HCCs might show *IDH1* mutations more often than known. Kipp et al. (2012) evaluated the histological features of cholangiocarcinomas with *IDH* mutations [6]. They demonstrated that poor differentiation or clear cell changes were associated with *IDH* mutations in cholangiocarcinomas. So, we decided to examine HCCs with clear cell changes. HCCs with a pseudoglandular pattern were added to our experiments after analyzing open-source data such as The Cancer Genome Atlas (TCGA) [9].

The aim of this study was to find specific subtypes of HCC with *IDH1* mutation. There has been no study of *IDH1* mutations in specific subtypes of HCC. HCCs in general showed no remarkable increase of *IDH1* mutations [4, 8, 10, 11]. We performed pyrosequencing for *IDH1* mutation analysis in clear cell HCCs and pseudoglandular HCCs. Only clear cell HCCs showed *IDH1* mutations.

## Methods

### Selection for specific subtypes of HCC from open-source data

The cBioPortal for Cancer Genomics (http://cbioportal.org) is an easy-to-use Web interface tool for exploring data from the large-scale cancer genomics projects, such as The Cancer Genome Atlas (TCGA) [12, 13]. It provided three studies of HCC, including RIKEN (Rikagaku Kenkyusho, Institute of Physical and Chemical Research, Japan) study (21 samples) [14], AMC (Asan Medical Center, Korea) study (231 samples) [15], and TCGA study (provisional, 442 samples). A query was submitted to inspect the *IDH1* status of these HCC samples.

The virtual slide images of TCGA HCCs were available on The Cancer Digital Slide Archive (http://cancer.digitalslidearchive.net) except those of AMC study [9]. The attached pathology reports were also reviewed to check any inconsistency between the initial pathologists and authors. HCCs with a pseudoglandular pattern were chosen for *IDH1* mutation analysis (described in detail in the “Results” section). In addition, HCCs with clear cell type were also selected for *IDH1* mutation study since Kipp et al. showed iCCs with clear cell change were significantly related to the increased *IDH1* mutation [6].

### Patients

**Fig. 1:**
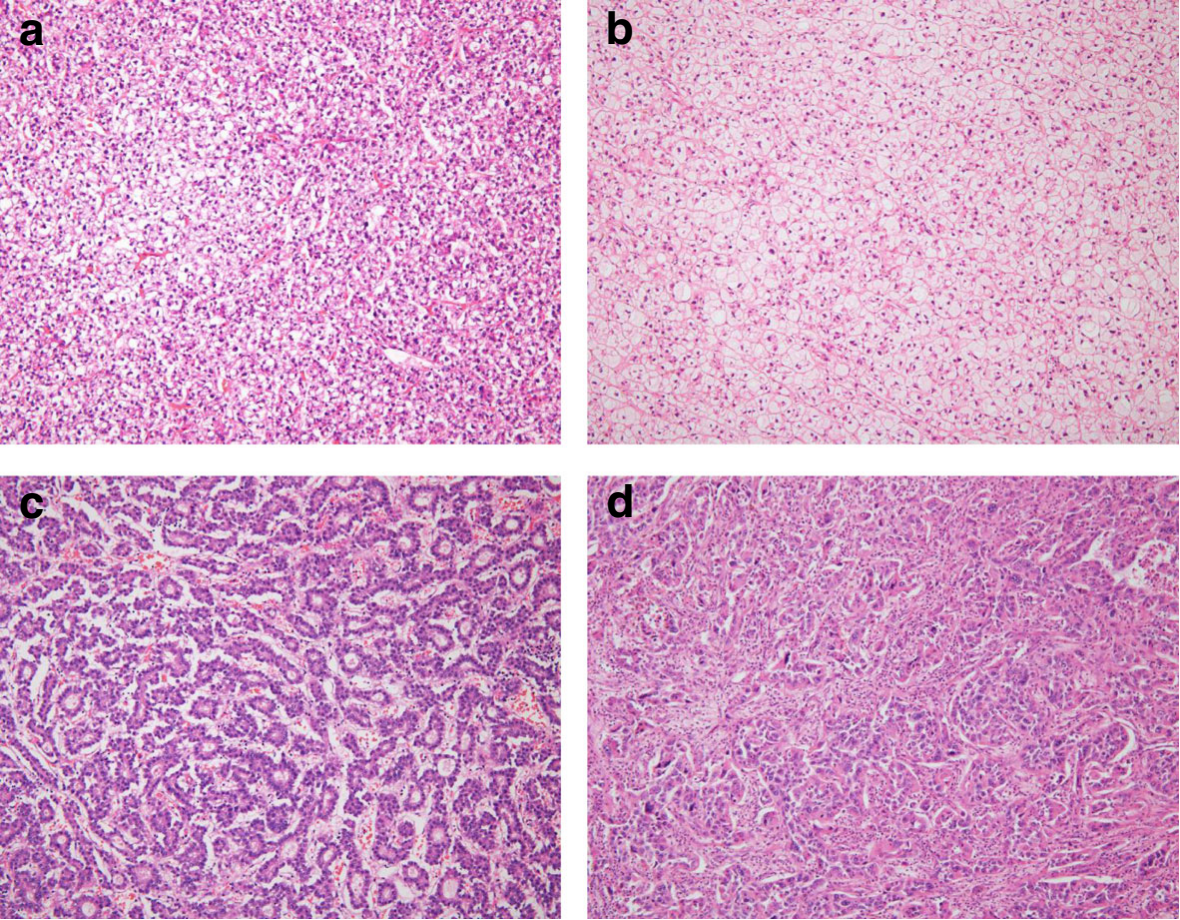
Representative microphotographs of selected samples (H&E stain, ×100). **a** Hepatocellular carcinoma with clear cell type (case no. HCC32). **b** Hepatocellular carcinoma with clear cell type (case no. HCC50). **c** Hepatocellular carcinoma with pseudoglandular pattern (case no. HCC33). **d** Mass-forming intrahepatic cholangiocarcinoma (case no. iCC19)

Primary HCCs were retrieved from surgically resected cases of the Pusan National University Yangsan Hospital between May 2009 and December 2014. The total number of resected HCCs was 371. From these 371 cases, “pseudoglandular” or “clear cell” HCCs were chosen from the electronic medical record system by reviewing the pathology reports and 36 cases were selected: 20 clear cell types, 13 pseudoglandular patterns, and 3 pseudoglandular patterns with clear cell type. HCCs with a clear cell type contained at least 70% of tumor cells with clear cytoplasm, and HCCs with a pseudoglandular pattern had at least 70% of pseudoglandular or acinar architecture (Fig. 1). HCCs with pseudoglandular pattern with clear cell type showed both pseudoglandular pattern and clear cell type, regardless of its proportion.

These were compared to HCCs with a trabecular pattern and classical (hepatic) cell type (14 cases) which were the most common HCC subtype. Additionally, iCCs were used as another control group. We found 46 iCC cases that fulfilled the established condition: mass-forming tumor within the liver (Fig. 1). Perihilar cholangiocarcinomas, also known as Klatskin tumors, were excluded in this study.

### Pyrosequencing

Slides stained with hematoxylin and eosin were reviewed to select samples that had adequate tumor lesions to perform DNA analyses. Two serial 10-μm sections of formalin-fixed paraffin-embedded (FFPE) tissues containing at least 70% of the tumor were cut, deparaffinized by xylene (55 °C for 3 min and 13,500 rpm for 3 min), and washed with 100% ethanol three times (13,500 rpm for 3 min each time). The resulting tissue pellets were dried at 55 °C for 10 min. DNA extraction was performed using a QIAamp DNA Mini Kit (Qiagen GmbH, Hilden, Germany), following the manufacturer’s protocol. The NanoDrop 2000 (Thermo Fisher Scientific, Waltham, MA, USA) spectrophotometer was used to measure the purity and concentration of DNA in the samples.

Purified DNA was amplified by PCR using primers specific to the *IDH1* codon 132 (Table 1). PCR was performed in a 26-μl total reaction volume containing 5 μl of the DNA template (2–10 ng), 1 μl of the forward primer (10 μmol/L), and 1 μl of the reverse primer (10 μmol/L). The PCR amplification protocol consisted of 95 °C for 15 min and 42 cycles of 95 °C for 20 s, 53 °C for 30 s, and 72 °C for 20 s, with a final extension for 5 min at 72 °C.

**Table 1:** Sequences used in analysis of *IDH1* mutation by pyrosequencing

Forward primer	
5′-GCTTGTGAGTGGATGGGTAAAA-3′	
Reverse primer	
5′-biotin-ATTGCCAACATGACTTACTTGATC-3′	
Sequencing primer	
5′-TGGGTAAAACCTATCATC-3′	
Dispensation order	
GATACGTAGCATGTCAT	
Sequence to analyze	
ATAGGTNGTCAT	

Pyrosequencing was performed on a Qiagen PyroMark Q24 system (Qiagen GmbH, Hilden, Germany) according to the manufacturer’s protocol. A mixture containing 10 μl of PCR product, 2 μl of Streptavidin Sepharose High Performance beads (GE Healthcare, Buckinghamshire, UK), 28 μl of water, and 40 μl of PyroMark binding buffer (Qiagen GmbH, Hilden, Germany) was agitated for 10 min at 1400 rpm to bind PCR products to the beads. The beads were then captured using a vacuum workstation (Qiagen GmbH, Hilden, Germany), washed in 40 ml of 70% ethanol for 5 s, denatured with 40 ml of denaturation buffer (Qiagen GmbH, Hilden, Germany) for 5 s, and washed in 50 ml washing buffer (Qiagen GmbH, Hilden, Germany) for 10 s. The beads were then released, and the purified DNA samples were annealed to the sequencing primer (Table 1) (0.3 μmol/L) in 25 μl of annealing buffer (Qiagen GmbH, Hilden, Germany) for 2 min at 80 °C and cooled for 10 min at room temperature.

The PyroMark Q24 was used for pyrosequencing using PyroMark Gold Q24 Reagents (Qiagen GmbH, Hilden, Germany). Pyrograms were manually interpreted and evaluated using the PyroMark Q24 software (Qiagen GmbH, Hilden, Germany). Unmethylated human DNA (Qiagen GmbH, Hilden, Germany) served as a negative control. The FFPE of a chondrosarcoma was used as a positive control which was verified using Sanger sequencing.

### Statistical analysis

Statistical analysis was carried out using the Statistical Package for the Social Sciences (SPSS) version 19 for MS-Windows. Two-sided Fisher’s exact test was performed to compare categorical variables, and Mann-Whitney test was used to compare discrete variables. Survival curves were drawn by a Kaplan-Meier method and compared using a log-rank test and Cox regression model for both univariate and multivariate analyses.

## Results

### *IDH1* status of 3 TCGA HCC studies

**Fig. 2:**
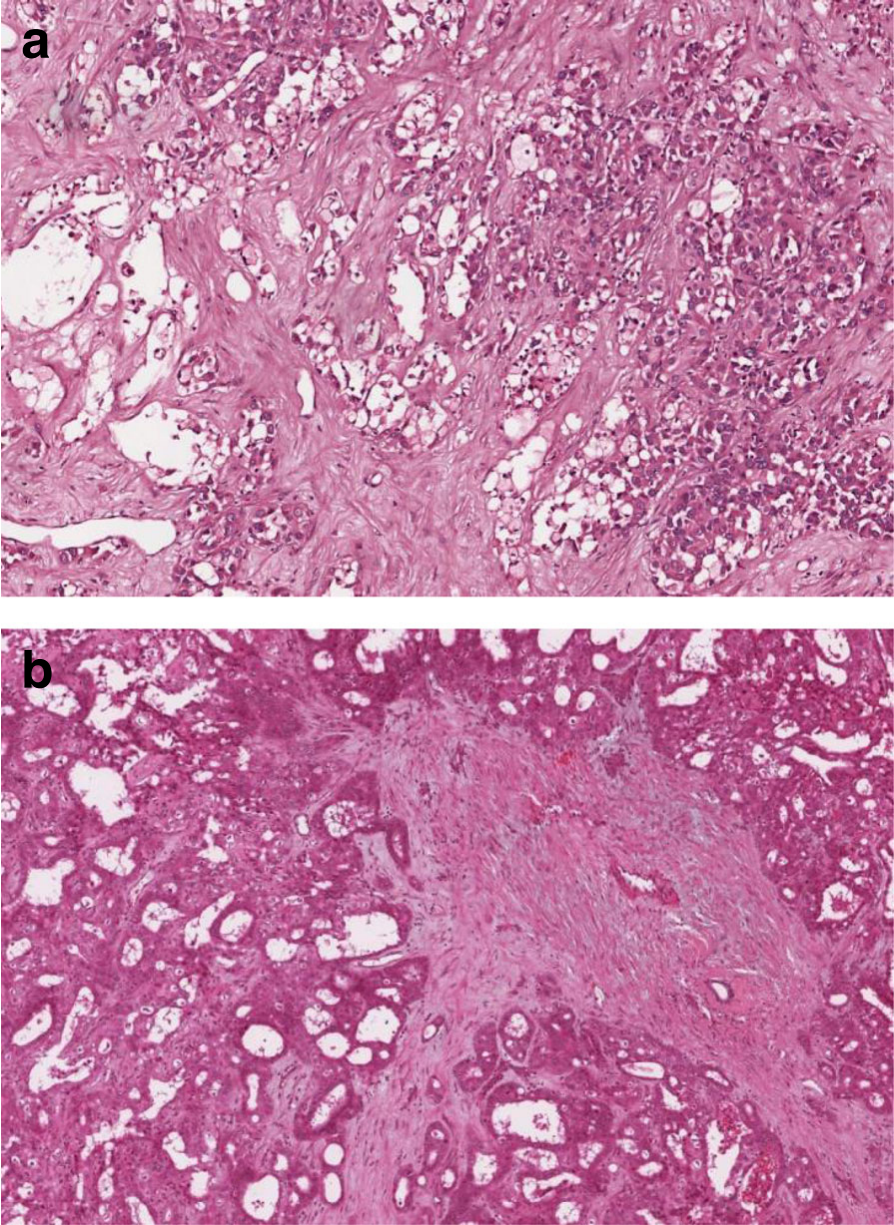
The captured virtual slide images of hepatocellular carcinomas with *IDH1* mutation (http://cancer.digitalslidearchive.net). The overall common patterns were pseudoglandular (described as adenoid, acinar, or tubular in its own pathology report) structure and sclerosing stroma. **a** Sample ID is TCGA-CC-5260-01; amino acid change, *IDH1* R132C. **b** Sample ID is TCGA-BC-A10Q-01; amino acid change, *IDH1* R132C

*IDH1* mutations were revealed in none (0%) of 21 RIKEN cases, in 1 (0.4%) of 231 AMC cases, and in 3 (1.6%) of 193 TCGA cases (Table 2). The remarkable histologic features of 3 TCGA HCCs with *IDH1* mutations were pseudoglandular pattern and sclerosing stroma with various differentiation grades (Fig. 2). No significant discrepancy was found between the initial pathologists and authors.

**Table 2:** The profile of *IDH1* mutations in hepatocellular carcinoma studies collected from cBioPortal website (http://cbioportal.org)

Gene	Sample ID	Cancer study	AA change	COSMIC	Allele frequency
*IDH1*	H090284	Liver (AMC)	R132L	4964	0.11
	TCGA-CC-5260-01	Liver (TCGA)	R132C	4964	0.29
	TCGA-BC-A10Q-01	Liver (TCGA)	R132C	4964	0.47
	TCGA-DD-A4NA-01	Liver (TCGA)	R132G	4964	0.46

*AA* indicates amino acid, *COSMIC* Catalogue of Somatic Mutations in Cancer, *AMC* Asan Medical Center, *TCGA* the Cancer Genome Atlas

### Clinical features

We studied 48 HCC patients, 36 men and 12 women, with an average age of 58.8 years (range 39–76 years). Out of the 48 patients, 30 (62.5%) had chronic hepatitis B virus (HBV) infection and 28 (58.3%) showed cirrhosis in the surrounding liver tissue (Table 3).

**Table 3:** Clinicodemographic data of intrahepatic cholangiocarcinomas (iCCs) and hepatocellular carcinomas (HCCs)

	Male	Female	Age (mean)	HBV	Cirrhosis
iCC	35 (76.1%)	11 (23.9%)	38–77 (61.9)	5 (10.9%)	2 (4.3%)
HCC	36 (75.0%)	12 (25.0%)	39–76 (58.8)	30 (62.5%)	28 (58.3%)
Total	71 (75.5%)	23 (24.5%)	38–77 (60.3)	35 (37.2%)	30 (31.9%)

iCC patients consisted of 35 men and 11 women (46 total), with an average age of 61.9 years (range 38–77 years). Out of the 46 patients, 5 (10.9%) had chronic HBV infection and 2 (4.3%) showed cirrhosis in the surrounding liver tissue (Table 3).

### *IDH1* mutation analysis by pyrosequencing

**Fig. 3:**
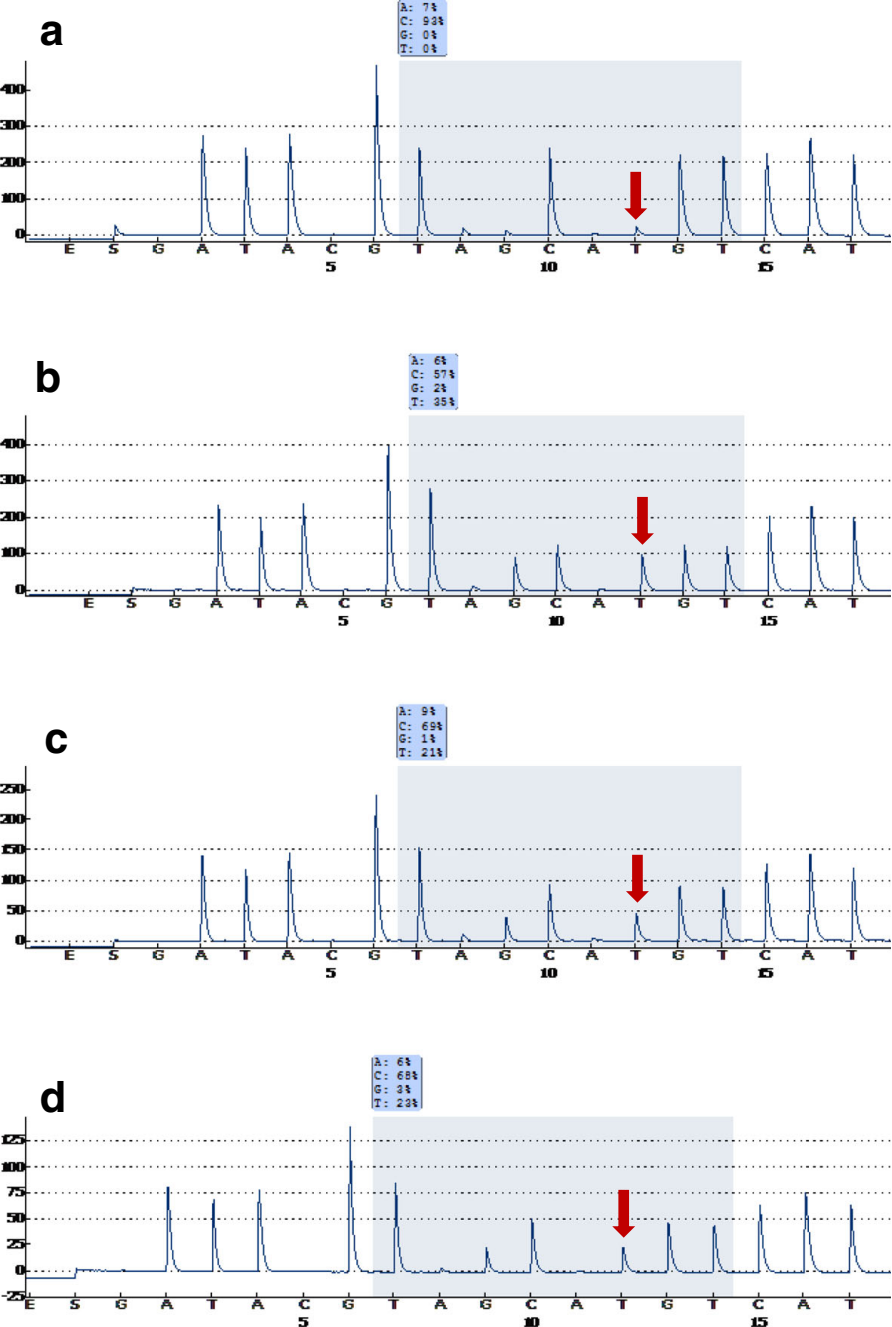
Pyrograms for *IDH1* codon 132. **a** Wild type (no mutation). **b** Positive control (a chondrosarcoma) (CGT>TGT, 35% frequency). **c** Case no. HCC32 (CGT>TGT, 21% frequency). **d** Case no. HCC50 (CGT>TGT, 23% frequency). (Histologic photographs of **c** and **d** are presented in Fig. 1)

The missense somatic mutation at codon 132 of the *IDH1* gene was detected in 8 cases (8.3%): 3 in iCCs (6.5%) and 5 in HCCs (10.4%). Differences in *IDH1* mutations between iCCs and HCCs were not statistically significant (*P* = 0.381) (Table 4). In all instances, an arginine to cysteine substitution (R132C) occurred due to a codon change of “CGT” to “TGT” (Fig. 3). A frequency value above 10% for the heterozygous allele TGT was determined to be a mutation since the wild type allele showed a 0% frequency value of TGT. In addition, 43 cases of 46 iCCs and 41 cases of 50 HCCs showed a 0% frequency of TGT. One of HCCs with a clear cell type showed an 8% frequency of TGT and three of HCCs (2 clear cell type and 1 pseudoglandular pattern) showed a 1% frequency of TGT.

**Table 4:** The proportion of *IDH1* mutations at codon 132 by pyrosequencing in iCCs and HCCs

Classifications		*IDH1* R132C mutations	
iCCs		3/46 (6.5%)	
HCCs	Total	5/48 (10.4%)	
	Clear cell type		5/20 (25.0%)
	Pseudoglandular pattern		0/13 (0.0%)
	Pseudoglandular pattern with clear cell type		0/3 (0.0%)
	Trabecular pattern with hepatic cell type		0/12 (0.0%)
		*P* = 0.381	*P* = 0.009

The genetic alterations were all CGT (arginine) to TGT (cysteine)

*IDH1*-mutant HCCs were all clear cell type. HCCs with a clear cell type were classified as a “clear cell group” and HCCs including a pseudoglandular pattern, trabecular pattern, or hepatic cell type were classified as a “non-clear cell group.” Differences in *IDH1* mutations between “clear cell group” and “non-clear cell group” were statistically significant (*P* = 0.009) (Table 4). Within HCC cases, clinical parameters such as sex, age, chronic HBV infection, or cirrhosis were not associated with *IDH1* mutations (Table 5).

**Table 5:** Characteristics of *IDH1*-mutated iCCs and HCCs

Case no.	Allele frequency	Sex	Age	HBV	Cirrhosis	Follow-up months	Alive	Metastasis/recurrence	Histologic grade^a^
iCC19	0.15	Male	54	−	−	9	No	−/+	3
iCC29	0.13	Female	70	−	−	4	No	−/−	2
iCC49	0.13	Male	38	+	−	4	No	+/−	2
HCC10	0.16	Female	47	−	+	3	No	−/−	2
HCC32	0.21	Male	64	+	−	16	Yes	−/−	1
HCC35	0.14	Female	67	−	+	2	No	−/+	3
HCC38	0.17	Male	51	+	+	0	No	−/−	1
HCC50	0.23	Male	76	−	+	2	NA	−/−	1

*LC* indicates liver cirrhosis, *NA* not available

^a^Histologic grade 1, well differentiated; grade 2, moderately differentiated; grade 3, poorly differentiated

### Survival analysis

Follow-up started since the operation day. Follow-up periods ranged between 4 days and 76 months. The patient with four follow-up days underwent liver transplantation and was expired due to postoperative pulmonary hypertension. This datum was omitted from survival analysis.

During the follow-up period, 19 patients (13 iCCs and 6 HCCs) became deceased, 24 patients (17 iCCs and 7 HCCs) developed metastasis, and 22 patients (10 iCCs and 12 HCCs) experienced local recurrence. The mean survival (95% confidence interval) was 46.5 months for iCC patients (37.8–55.1 months) and 66.8 months for HCC patients (59.9–73.6 months). There was statistically significant difference between survival times of iCC and HCC patients (*P* = 0.037).

**Fig. 4:**
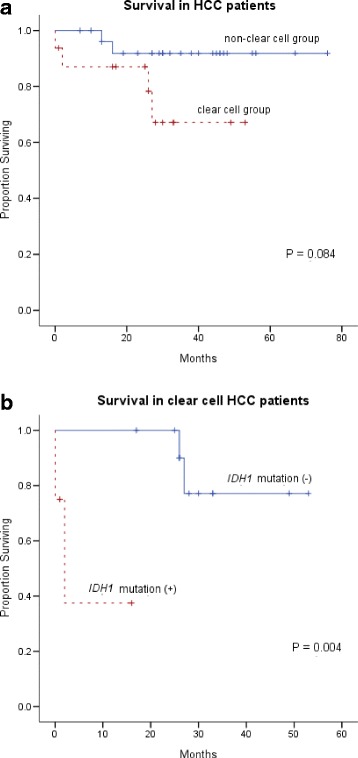
Survival analysis of HCC patients. **a** Clear cell group showed lower overall survival rate than non-clear cell group (*P* = 0.089). **b** In clear cell group, the overall survival rate is lower in patients with *IDH1* mutation (*P* = 0.004)

Since four pT3 HCCs were all censored due to loss to follow-up and there was no pT4 HCC, only pT1 and pT2 stages of HCCs were included in HCC survival analysis. (One of pT3 HCCs was *IDH1* mutant.) For 43 cases of HCC, the estimated proportional hazard ratios were 79.546 (95% confidence interval, 4.981~1270.295; *P* = 0.002) for the presence of *IDH1* mutation and 18.447 (95% confidence interval, 2.032~167.498; *P* = 0.010) for pT2 stage. Multivariate analysis showed the presence of *IDH1* mutation was associated with poor prognosis of HCC patients, independently of tumor stage, sex, age at diagnosis, HBV infection, and cirrhosis. Univariate analysis showed that a clear cell type was not associated with HCC patients’ survival (*P* = 0.084) and shorter survival times for clear cell HCC patients was associated with an *IDH1* mutation (*P* = 0.004) (Fig. 4).

## Discussion

A novel finding was that HCCs with *IDH1* mutation belonged to “clear cell group” without exception. The finding that not a single case of pseudoglandular HCCs was *IDH1* mutant was unexpected since all the three TCGA HCCs with *IDH1* mutation had a pseudoglandular pattern. This discrepancy might be generated by the difference in the number of cases. We made experiments on 48 HCCs while TCGA study had 193 HCCs, four times more cases. Another possible explanation is that the sclerosing stroma could be better correlated with *IDH1* mutations than the pseudoglandular pattern. Although we had considered the extent of stromal deposition as a candidate morphological factor for *IDH1* mutations, the stromal change was not included in the analysis. Meanwhile, the result that HCCs with both trabecular pattern and hepatic cell type showed no *IDH1* mutation was consistent with our expectations deduced from the previous studies. To make our results more reasonable, the experiments should be done for additional consecutive HCC cases containing variable morphological factors.

A “clear cell type” classification of HCCs seemed to associate with a decreased survival time, but not statistically verified (Fig. 4). Multiple studies produced different results about the association of a clear cell type with HCC patient survival. Studies conducted in Hong Kong and China found that the clear cell ratio in HCC samples was proportional to a better prognosis [16–18]. On the contrary, a study performed in Japan demonstrated that clear cell type HCCs did not have a significantly different prognosis, when compared to non-clear cell HCCs [19]. Additionally, a French study found that clear cell type HCC patients had a similar prognosis as other HCC patients [20]. Thus, the clinical significance of clear cell type HCCs can be revised and modified accordingly.

Clear cell carcinomas can present in any organ throughout the body. Among these, the urogenital organs such as the kidneys or ovaries are the most common. Studying *IDH1/2* mutations in clear cell renal cell carcinomas (RCCs) demonstrated that *IDH1* is mutated in only 4 of 833 cases (0.5%) and no *IDH2* mutations [12, 13]. There have been no other studies reporting *IDH1/2* mutations in clear cell RCCs. Clear cell RCCs are less aggressive than RCCs with a granular cytoplasm; this difference seems to be partly caused by nuclear grade effects [21]. Meanwhile, there are no studies determining *IDH1/2* mutations in clear cell ovarian carcinomas, which have a poorer prognosis than serous ovarian adenocarcinomas [22].

*IDH1* mutant HCC patients in the present study were linked with poor survival rates. Glioma patients, however, with *IDH1* codon 132 mutation experienced a better prognosis [23]. In the case of gliomas, a codon change from CGT to “CAT” (c.395G>A and p.Arg132His) is the most frequent type of *IDH1* mutation [24]. However, the *IDH1* mutation profile in this study was different; the codon change was from CGT to TGT (c.394C>T and p.Arg132Cys). Gravendeel et al. (2010) validated that non-R132H mutation groups were segregated from R132H mutation groups, both histologically and molecularly, in gliomas [25]. Although this segregation could not account for the differences in patient’s survival times, the effects of R132H and non-R132H mutations on carcinogenesis need to be further investigated.

Clear appearance of the cytoplasm is due to dissolving of abundant intracellular glycogen and lipid during tissue preparation. The liver is a highly active organ in the metabolism of lipid. Lipid metabolism is regulated by IDH1 which is activated by sterol regulatory element-binding protein (SREBP) [26, 27]. SREBP directly mediates cholesterol and fatty acid synthesis and indirectly does the same by leading to the expression of *IDH1*. IDH1 provides NADPH, a driving force of reductive reaction such as lipogenesis. SREBP induces expression of mutant IDH1 as well as the wild type one [28]. Hence, it could be inferred that mutant *IDH1* is associated with aberrant lipogenesis and clear cell appearance in HCCs.

*IDH1* R132C mutations in HCCs (10.4%) occurred more frequently than in iCCs (6.5%) (Table 4). The overall frequency of *IDH1* mutation rate in HCCs is higher than that of TCGA study (1.6%). TCGA HCCs with *IDH1* mutation have relatively high allele frequency values: 0.29, 0.46, and 0.47 (Table 2). On the other hand, the present study showed low allele frequency values in HCCs with *IDH1* mutation, from 0.13 to 0.23 (Table 5). We presume that (1) the mutation allele frequency of *IDH1* in HCCs is too low to be detected by conventional method, and (2) it could be linked to late phase of HCC development.

First, the pyrosequencing is known to have high sensitivity. It has been proven experimentally through the detection of *IDH1/2* mutations in gliomas [29]. We speculate that our results are due to the sensitivity of the pyrosequencing assay and not caused by false positives.

Second, the somatic mutations in the late phase (branch mutation) of cancer development have not enough time to be stacked and show lower allele frequency, while the mutations in the early phase (trunk mutation) accumulate and have higher allele frequency [30]. This presumption can also explain that HCC patients with *IDH1* mutation showed poor survival (Fig. 4) despite its wide confidence interval. Contrastingly, *IDH1* mutation in glioma develops in early phase and is associated with easy detection and good prognosis [23]. Thus, even the same mutations in the same gene may present different expression values and variable clinical significance, depending on when it is activated [31].

## Conclusions

*IDH1* R132C mutation is seen in clear cell HCC but not in other variants of HCC. From this result, we confirm that morphological features of tumors reflect molecular alterations. While clear cell type is not associated with HCC patient survival, *IDH1* mutation is relevant for shorter survival times in clear cell HCCs.

## Abbreviations

AMC: Asan Medical Center
FFPE: Formalin-fixed paraffin-embedded
HBV: Hepatitis B virus
HCC: Hepatocellular carcinoma
iCC: Intrahepatic cholangiocarcinoma
*IDH1*: Isocitrate dehydrogenase 1
NADPH: Nicotinamide adenine dinucleotide phosphate
RCC: Renal cell carcinoma
RIKEN: Rikagaku Kenkyusho
SREBP: Sterol regulatory element-binding protein
TCGA: The Cancer Genome Atlas
